# Future research into the treatment of vitiligo: where should our priorities lie? Results of the vitiligo priority setting partnership

**DOI:** 10.1111/j.1365-2133.2010.10160.x

**Published:** 2011-03

**Authors:** V Eleftheriadou, ME Whitton, DJ Gawkrodger, J Batchelor, J Corne, B Lamb, S Ersser, J Ravenscroft, KS Thomas, on behalf of the vitiligo priority setting partnership

**Affiliations:** Centre of Evidence Based Dermatology, University of NottinghamNottingham NG7 2NR, U.K.

## Abstract

**Background:**

Vitiligo is the most frequent depigmentation disorder of the skin and is cosmetically and psychologically devastating. A recently updated Cochrane systematic review ‘Interventions for vitiligo’ showed that the research evidence for treatment of vitiligo is poor, making it difficult to make firm recommendations for clinical practice.

**Objectives:**

To stimulate and steer future research in the field of vitiligo treatment, by identifying the 10 most important research areas for patients and clinicians.

**Methods:**

A vitiligo priority setting partnership was established including patients, healthcare professionals and researchers with an interest in vitiligo. Vitiligo treatment uncertainties were gathered from patients and clinicians, and then prioritized in a transparent process, using a methodology advocated by the James Lind Alliance.

**Results:**

In total, 660 treatment uncertainties were submitted by 461 participants. These were reduced to a list of the 23 most popular topics through an online/paper voting process. The 23 were then prioritized at a face-to-face workshop in London. The final list of the top 10 treatment uncertainties included interventions such as systemic immunosuppressants, topical treatments, light therapy, melanocyte-stimulating hormone analogues, gene therapy, and the impact of psychological interventions on the quality of life of patients with vitiligo.

**Conclusions:**

The top 10 research areas for the treatment of vitiligo provide guidance for researchers and funding bodies, to ensure that future research answers questions that are important both to clinicians and to patients.

Vitiligo is the most common chronic depigmentation disorder affecting around 0·5%^1^,^2^ of the world population. It is cosmetically and psychologically devastating,^3^ and can result in low self-esteem, poor body image and difficulties in sexual relationships.^4^–^7^ The causes of vitiligo are poorly understood and treatment is often unsatisfactory.^8^

Sixty-eight treatments for vitiligo have been evaluated in clinical trials over the last 43 years. However, due to the small numbers of participants and heterogeneity of design of trials to date, it is difficult to make firm recommendations for clinical practice.^9^ Indeed, in the face of so many treatment options and with so little information regarding their relative efficacy, it is difficult to identify which clinical trials are most important and timely.

In order to address this concern, this project was established with the aim of helping to identify the following. (i) Which interventions should be evaluated? (ii) What are the most important topics to patients and clinicians? (iii) Could these topics be answered by clinical research?

It is increasingly recognized that patients and healthcare professionals have a key role to play in identifying important areas for research. The James Lind Alliance (JLA) is a Department of Health and Medical Research Council funded initiative, which has been established to bring patients and clinicians together in ‘priority setting partnerships’ (PSPs) to identify and prioritize the unanswered questions that they agree are most important.^10^ The pharmaceutical and medical technology industries and academia play an essential role in developing new treatments.^11^ However, the priorities of industry and academics are not necessarily the same as those of patients and clinicians. For this reason many areas of potentially valuable research are neglected. Therefore it is essential that researchers and funding bodies are aware of the needs of patients and clinicians.

This was the first PSP in the field of dermatology and the third of its kind to have been convened by the JLA. Previous partnerships have been conducted in the fields of asthma^11^ and urinary incontinence.^12^

All the uncertainties identified by the PSPs are added to the Database of Uncertainties about the Effects of Treatments (DUETs) in order to provide reference for funding bodies and researchers. It is known that the research funding bodies in the U.K. systematically scan important research resources to identify evidence gaps and make recommendations for research. This includes Cochrane systematic reviews and more recently DUETs.

DUETs has been established in the U.K. to publish uncertainties about the effects of treatment which cannot currently be answered by referring to reliable up-to-date systematic reviews of existing research evidence.^13^ A treatment uncertainty exists when ‘no up-to-date systematic review exists, or up-to-date systematic reviews show that uncertainty continues’,^14^ i.e. more research needs to be done to establish the effectiveness and safety of an existing or innovative intervention.

## Materials and methods

The vitiligo PSP was coordinated at the Centre of Evidence Based Dermatology, University of Nottingham, with numerous stakeholders from professional organizations and patient support groups. The aim of the vitiligo PSP was to reduce the number of uncertainties surrounding the treatment of this condition and to steer future research to questions of importance both to people living with the disease and to people treating the disease.

**Fig 1:**
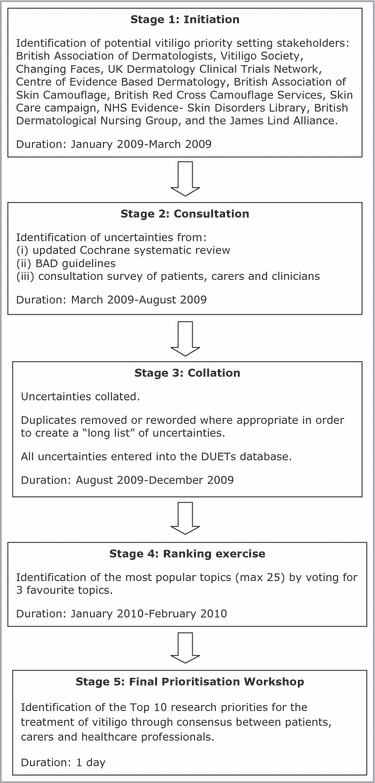
Summary of methods used by the vitiligo priority setting partnership. BAD, British Association of Dermatologists; DUETs, Database of Uncertainties about the Effects of Treatments.

The vitiligo PSP adopted the methods advocated by the JLA^11^ which were refined to meet the needs of this particular PSP.^11^ The vitiligo PSP had five stages (see Fig. 1 for a summary of the vitiligo PSP methodology).

### Stage 1: Initiation

The aim of this stage was to establish the vitiligo PSP by raising awareness, and identifying and engaging potential stakeholders.

Organizations approached during this stage were professional bodies and patient support groups: British Association of Dermatologists (BAD), UK Dermatology Clinical Trials Network (UK DCTN), NHS Evidence – Skin Disorders, Cochrane Skin Group, British Dermatological Nursing Group, Changing Faces, British Red Cross Camouflage Service, Skin Care Campaign, Primary Care Dermatology Society, Vitiligo European Task Force, British Association of Skin Camouflage and Vitiligo Society. Individual researchers, dermatologists, specialist nurses and psychologists with a special interest in vitiligo were also informed. Our research group, called the Steering Group, included 12 members with knowledge and interest in vitiligo. (For details on the Steering Group members please see Acknowledgments section).

### Stage 2: Consultation

The aim of this stage was to collect treatment uncertainties.

An online and paper survey was undertaken that encouraged patients and clinicians to submit their questions about the treatment of vitiligo. Paper copies of the questionnaire were sent to the Vitiligo Society (*n* = 1268) and to the BAD (*n* = 835). E-mails were sent to members of the UK DCTN (*n* = 500), and details of the project (with links to the online survey) were advertised on the websites and in the newsletters of the relevant organizations listed above.

Additional treatment uncertainties were identified from existing sources of current evidence: the updated Cochrane systematic review ‘Interventions for vitiligo’^9^ and the BAD guideline for diagnosis and management of vitiligo.^15^

### Stage 3: Collation

The aim of this stage was to create a ‘long-list’ of uncertainties by collating, refining submitted uncertainties and rewording similar questions. Questions on the aetiology, the natural history and prevention of the disease (non-treatment uncertainties) were excluded at this stage. Each collated uncertainty represented a broad area for research, rather than focusing on a specific research question. For example, the refined uncertainty ‘How effective is ultraviolet B therapy when combined with creams or ointments in treating vitiligo?’ includes combination of narrowband ultraviolet B with topical agents such as corticosteroids, calcineurin inhibitors, vitamin D analogues etc. This was necessary in order to reduce the list of uncertainties to a manageable number.

### Stage 4: Ranking exercise (Interim prioritization exercise)

The aim of the ranking exercise was to create a ‘short-list’ of uncertainties and to reduce their number to no more than 25. As the majority of participants from the consultation stage expressed a willingness to engage in the process further, the ranking exercise included all people who gave contact details during the consultation. It was also advertised on the websites and in the newsletters of relevant organizations, as per the consultation stage. In addition, advertisements and articles were placed in the Voice magazine for black and ethnic minorities, the British Dermatological Nursing Group magazine^16^ and the bulletin of the Primary Care Dermatology Society^17^ to target specific groups that had been under-represented during the consultation stage.

Participants were asked to vote for their three favourite topics (three individual votes) online (http://www.vitiligostudy.org.uk) or using paper questionnaires by downloading them from our website or contacting the research team directly. The order in which uncertainties appeared on the survey was randomized in order to guard against response bias.

### Stage 5: Final Prioritization Workshop

The aim of this final stage was to identify the top 10 most important treatment uncertainties for vitiligo by creating consensus through a face-to-face workshop of healthcare professionals and patients.

Participants of previous stages of the vitiligo PSP attended this workshop. Efforts were made to ensure that equal numbers of patients and healthcare professionals attended. The workshop was a full-day event, held at the London offices of the BAD on 25 March 2010.

Further details of the methods used during the vitiligo PSP are outlined in the James Lind Alliance guidebook (http://www.jlaguidebook.org/).

### Ethics

This project was approved by the Medical School Research Ethics Committee, University of Nottingham, U.K, Ethics Reference No. G/2/2009.

### Statistical methods

We aimed for a minimum of 100 participants in the consultation and the ranking exercise and for 20 participants for the final prioritization workshop. This sample size was estimated on the basis of previous JLA PSPs,^18^ and determined by the time frame available for the vitiligo PSP.

Data from all stages were stored and analysed in Access 2007 and processed by the Steering Group members.

## Results

### Stages 2 and 3: Consultation and collation

Of the 2303 surveys circulated, 461 (20%) were returned. This resulted in 1427 questions about vitiligo. Non-treatment questions (*n* = 767), about the natural history of vitiligo, its aetiology and prevention, were excluded.

The response rate for members of the Vitiligo Society was 24% (307/1268) and for BAD/UK DCTN members was 14% (119/835). Sixty-six per cent of responses (302/461) were from patients, 31% (142/461) were from healthcare professionals, and 3% were from other sources. More women responded than men (53% women, 30% men, 17% did not specify), and most were aged 30–60 years (8% < 30 years, 50% 30–60 years, 25% > 60 years, 17% did not specify).

Overall, 660 uncertainties that specifically related to the treatment of vitiligo were gathered during the consultation stage. Thirty-one per cent were from healthcare professionals (206/660), 48·5% were from patients (320/660) and 20·5% were unknown (134/660). An additional 58 treatment uncertainties were identified from the BAD guideline and the updated Cochrane systematic review. The resulting 718 uncertainties were refined into a ‘long-list’ of 93 treatment uncertainties, which were used for the ranking exercise.

### Stage 4: Ranking exercise (interim prioritization exercise)

In total, 230 people (patients 72%, health care professionals 23%, did not specify 5%) responded to the ranking exercise, submitting 638 individual votes. Each participant could vote for up to three of their favourite uncertainties. Nineteen paper voters were excluded as they submitted more than three favourite topics. The number of votes per uncertainty ranged from 49 to 0 (median 5).

The demographic characteristics of participants in the ranking exercise were broadly similar to those in the consultation stage (63% were women, and 55% were aged between 30 and 60 years). Of those who specified their ethnicity (*n* = 127), 42% were white and 12·6% were from black and ethnic minorities.

As more patients participated in the ranking exercise than healthcare professionals, the Steering Group considered the ranked priorities of patients and healthcare professionals separately.

At the end of this stage, a short-list of 23 uncertainties was identified for the final prioritization workshop.

### Stage 5: Final Prioritization Workshop

The workshop was attended by 47 people: 21 were patients or patients’ representatives, and 16 were healthcare professionals (see Acknowledgments section for more details on the attendees).

Feedback following the workshop showed that all attendees were either very satisfied or satisfied with the top 10 uncertainties identified on the day and the vitiligo PSP was announced the most successful PSP so far by the JLA.

The top 10 treatment uncertainties for vitiligo as defined by clinicians and patients were:

1. How effective are systemic immunosuppressants in treating vitiligo?
2. How much do psychological interventions help people with vitiligo?
3. Which treatment is more effective for vitiligo: light therapy or calcineurin inhibitors?
4. How effective is ultraviolet B therapy when combined with creams or ointments in treating vitiligo?
5. What role might gene therapy play in the treatment of vitiligo?
6. How effective are hormones or hormone-related substances that stimulate pigment cells (melanocyte-stimulating hormone analogues, afamelanotide) in treating vitiligo?
7. Which treatment is more effective for vitiligo: calcineurin inhibitors or steroid creams/ointments?
8. Which treatment is more effective for vitiligo: steroid creams/ointments or light therapy?
9. How effective is the addition of psychological interventions to patients using cosmetic camouflage for improving their quality of life?
10. How effective is pseudocatalase cream (combined with brief exposure to ultraviolet B) in treating vitiligo?

In addition, two treatment uncertainties were suggested as ‘ones to watch’, as these interventions were still in an early investigative stage.

11 How effective is piperine (black pepper) cream in treating vitiligo?
12 What role might stem cell therapy play in treating vitiligo?

Finally, important recurring themes for researchers to consider when developing future trials emerged and are summarized below (Table 1). These themes covered general issues that were relevant to all therapeutic interventions for vitiligo.

**Table 1:** General themes to be considered when designing future vitiligo trials

	General theme
1	Which treatments are effective and safe for children?
2	Do treatment success rates differ according to the site(s) affected, or the gender/age/ethnicity/skin phototypes of patients?
3	What are the long-term outcomes of treatments for vitiligo (especially side-effects)?
4	What is the optimal duration and optimal timing for treatments of vitiligo?
5	What is the optimal maintenance regimen in order to prevent relapse?
6	Interventions for segmental vitiligo

**Fig 2:**
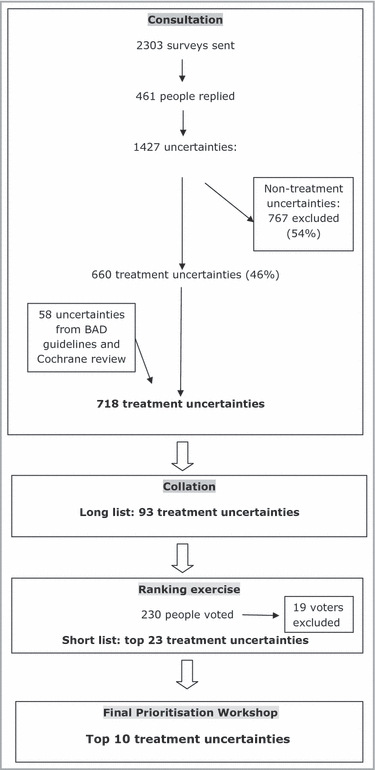
Summary of the results of the vitiligo priority setting partnership. BAD, British Association of Dermatologists.

Figure 2 presents a summary of the results of the vitiligo PSP.

## Discussion

Vitiligo has traditionally been given a relatively low priority in the dermatology research agenda, as shown by the number and quality of studies on vitiligo to date.^9^ The updated systematic review^9^ is helpful in identifying many important research gaps for clinical trials, but these have come largely from the research community, and may not reflect the questions that patients and clinicians have.

### Implication for research

The identified uncertainties provide a steer for future research activity by guiding researchers and funding bodies to questions of importance to patients and healthcare professionals. All of the uncertainties have been added to DUETs, and are freely available at http://www.library.nhs.uk/DUETs/Default.aspx. At this point it is important to remember that the vitiligo PSP aims to identify ‘treatment uncertainties’. These are then used as reference to inform future ‘research questions’ as developed by individual research teams. It is entirely possible that one treatment uncertainty will result in several related research questions.

Based on the most important treatment uncertainties for vitiligo identified, we recommend:

1. More research on the effectiveness and safety of systemic immunosuppresants for the treatment of vitiligo such as methotrexate or ciclosporin. Research in this field would potentially contribute to our knowledge about the aetiology of the disease, which is believed to have a strong autoimmune component^19^,^20^ (uncertainty 1).
2. Evaluation of currently available and widely used treatments such as topical corticosteroids and calcineurin inhibitors in a ‘head-to-head’ randomized controlled trial (uncertainty 7).
3. Evaluation of the effectiveness and safety of narrowband ultraviolet B. More detailed information is needed to answer questions such as ‘should the first line treatment for vitiligo be topical agents (topical steroids or calcineurin inhibitors) or more aggressive intervention such as narrowband ultraviolet B?’ A factorial trial design could evaluate all three treatment options in one trial (uncertainties 3 and 8).
4. Evaluation of psychological interventions by conducting a systematic review of the current literature, and substantial pilot/exploratory qualitative work prior to progressing to a full randomized controlled trial. Together with active treatments, psychological interventions are believed to be of great importance. More evidence is needed to establish the role of psychological support as monotherapy (uncertainty 2), as well as in combination with other treatments for vitiligo (uncertainty 9).
5. Evaluation of innovative treatments such as afamelanotide and pseudocatalase, which seem to be important and promising both to clinicians and to patients (uncertainties 6 and 10).
6. Evaluation of narrowband ultraviolet B combination therapies with topical agents, which also reflects the current research trend, shown by the Cochrane systematic review that combination treatments seem to be more effective than monotherapies^9^ (uncertainty 4).
7. More research to be done into the pathophysiology and the aetiology of the disease based on the great interest expressed by clinicians and patients on exploration of potential effectiveness of gene therapy and stem cells (uncertainty 5 and ‘one to watch’ uncertainty 12).

To conclude, we wish to note that by recommending the above we are not commenting on the legitimacy of the interventions that have been prioritized, but are reporting what clinicians and patients identified as important research topics in order to meet their needs.

### Reflections on the process

One might argue that the response rate for the vitiligo PSP was rather low (consultation stage response rate 20%); however, the number of participants by far exceeded our expectations based on previous PSPs convened by the JLA. This was due mainly to our collaboration and networking with the UK DCTN and the Vitiligo Society. We believe that the innovative and unusual nature of this project means that it is inappropriate to apply the same criteria for the response rate of this PSP as for other surveys. Indeed, the vitiligo PSP far exceeded our original sample size estimates for the number of participants at each stage, and had approximately double the number of participants who took part in previous PSPs.^18^

In order to inform future PSPs, it is helpful to present some of the key challenges that we faced as two recommendations.

**Recommendation 1**. We recommend that information about the existing research evidence for the different treatments is presented in a patient-friendly format at the beginning of the PSP. This would allow all participants to engage in the process more effectively, regardless of their background, experience or levels of expertise.
**Recommendation 2**. Most of the uncertainties (during consultation) were broad and nonspecific, or did not specify the comparator, the duration of treatment or the population, when others from the research community were very focused. For that reason, we recommend keeping the uncertainties as broad as possible to allow flexibility but sufficiently narrow to ensure the question is meaningful. Therefore we have developed a standard format for different types of questions submitted (Table 2).

**Table 2:** Structure used to refine the questions into indicative uncertainties

Type of question	Format of question
Effectiveness of a single treatment	How effective is [treatment X] in treating vitiligo?
One treatment *compared with* another	Which treatment is more effective in treating vitiligo: [treatment X or treatment Y]?
One treatment *combined with* another	How effective is [treatment X] when combined with [treatment Y] in treating vitiligo?
Management of the disease, rather than ‘treatment’ (e.g. camouflage or psychological interventions)	How much does [treatment X] help patients with vitiligo?
Speculative treatments not yet on the market (e.g. gene therapy, stem cell therapy)	What role might [treatment X] play in the treatment of vitiligo?

### Implementation of the results

The next step of our research group is to conduct a feasibility study on one of the top 10 uncertainties by working with the UK DCTN (http://www.ukdctn.org/home/).

We are hopeful that by publishing this list of important treatment uncertainties, we will prompt other research groups and pharmaceutical companies to take a fresh look at vitiligo research and the needs of patients with vitiligo and to bring unity to the international efforts into the treatment of vitiligo.

Finally, we would recommend that researchers continue to work with patients and clinicians in meaningful partnerships in developing their future research activity, in line with current guidelines.^21^

**What's already known about this topic?**

- Sixty-eight treatments for vitiligo have been evaluated in clinical trials over the last 43 years. However, due to the small numbers of participants and heterogeneity of design of trials to date, it is difficult to make firm recommendations for clinical practice.
- It is increasingly recognized that patients and healthcare professionals have a key role to play in identifying important areas for research. The pharmaceutical and medical technology industries and academia play an essential role in developing new treatments. However, the priorities of industry and academics are not necessarily the same as those of patients and clinicians. For this reason, many areas of potentially valuable research are neglected.

**What does this study add?**

- The research areas identified provide a steer for future research activity by guiding researchers and funding bodies to questions of importance to patients and healthcare professionals. There is a great need for better evaluation of the currently available and widely used treatments, such as topical corticosteroids, calcineurin inhibitors and phototherapy in a ‘head-to-head’ randomized controlled trial.
- Together with active treatments, psychological interventions are of great importance to patients and clinicians. More evidence is needed to establish the role of psychological support as monotherapy, as well as in combination with other treatments for vitiligo.
